# Morning and evening behavior in children and adolescents treated with atomoxetine once daily for Attention-Deficit/Hyperactivity Disorder (ADHD): Findings from two 24-week, open-label studies

**DOI:** 10.1186/1753-2000-3-5

**Published:** 2009-02-09

**Authors:** Peter M Wehmeier, Ralf W Dittmann, Alexander Schacht, Karin Helsberg, Gerd Lehmkuhl

**Affiliations:** 1 Lilly Deutschland, Medical Department, Bad Homburg, Germany; 2 Department of Child and Adolescent Psychiatry, Central Institute of Mental Health Mannheim, University of Heidelberg, Heidelberg, Germany; 3 Department of Child and Adolescent Psychiatry, University of Cologne, Cologne, Germany

## Abstract

**Background:**

The impact of once daily atomoxetine treatment on symptoms in children and adolescents with ADHD may vary over the day. In order to capture such variations, two studies were undertaken in children and adolescents with ADHD using two instruments that capture morning and evening behavior and ADHD-related difficulties over the day. This secondary measure analysis builds on two primary analyses that were conducted separately for children and adolescents and also published separately.

**Methods:**

In two open-label studies, ADHD patients aged 6–17 years (n = 421), received atomoxetine in the morning (target-dose 0.5–1.2 mg/kg/day) for up to 24 weeks. Morning and evening behavior was assessed using the investigator-rated Weekly Rating of Evening and Morning Behavior (WREMB-R) scale. ADHD-related difficulties at various times of the day (morning, during school, during homework, evening) were assessed using the Global Impression of Perceived Difficulties (GIPD) scale, rated by patients, parents and physicians. Data from both studies were combined for this secondary measure analysis.

**Results:**

Both WREMB-R subscores decreased significantly over time, the evening subscore from 13.7 (95% CI 13.2;14.2) at baseline to 8.0 (7.4;8.5) at week 2, the morning subscore from 4.3 (4.0;4.5) to 2.4 (2.2;2.6). Scores then remained stable until week 24. All GIPD items improved correspondingly. At all times of the day, patients rated ADHD-related difficulties as less severe than parents and physicians.

**Conclusion:**

These findings from two open-label studies suggest that morning and evening behavior and ADHD-related difficulties in the mornings and evenings improve over time with once daily atomoxetine treatment.

## Background

Attention-deficit/hyperactivity disorder (ADHD) affects 3–7% of school-age children and is characterized by inattention, impulsivity, and hyperactivity [1]. ADHD is usually associated with significant impairment of cognitive and psychosocial functioning [2,3] and can have a significant impact on the emotional well-being [4-6] and the quality of life (QoL) of both patients and their families [7-12].

Psychostimulants and behavioral therapy are known to be effective in the treatment of ADHD, as reported in the MTA study [13]. Since the introduction of various long-acting ADHD medications, interest in the efficacy profile of these compounds over the day and the possibility of once-daily dosing has increased [14-18]. Depending on the delivery profile of the various compounds, the exposure to stimulants such as methylphenidate may vary over the day, potentially resulting in declining efficacy towards the evening hours [19]. Thus, the impact of ADHD medications on core symptoms of ADHD over the day, on emotional well-being and QoL of patients is of considerable interest both to clinicians and researchers.

The non-stimulant atomoxetine is one of several long-acting treatment options for ADHD [20]. In contrast to the stimulants, atomoxetine is a selective norepinephrine reuptake inhibitor [21]. Efficacy and tolerability of atomoxetine in children and adolescents have been demonstrated in a number of randomized, placebo-controlled trials (for an overview and meta-analysis, see [22]. Furthermore, several studies have demonstrated a positive effect of atomoxetine on morning and evening behavior [17,23], emotional well-being [24,25], ADHD-related difficulties [26] and QoL [27-29], in children and adolescents with ADHD. In many of these studies, scales and questionnaires such as the ADHD Rating Scale (ADHD-RS) [30,31], the Clinical Global Impression (CGI) [32,33], the Child Health Questionnaire (CHQ) [34,35] or the Child Health and Illness Profile, Child Edition (CHIP-CE) [36] have been applied. These scales are useful when assessing ADHD symptoms, functional outcome or quality of life. However, they do not capture ADHD-related problems at various times of the day, which is important, as these may vary over the day [17,23]. To fill this gap, several scales assessing ADHD-related difficulties over the day have been developed. The Daily Parent Rating of Evening and Morning Behavior – Revised (DPREMB-R) scale [17,23,37] or the adapted Weekly Rating of Evening and Morning Behavior – Revised (WREMB-R) scale [38], and the Global Impression of Perceived Difficulties (GIPD) scale [39] are such instruments that allow the assessment of ADHD-related difficulties over the day.

The GIPD and WREMB-R were used in two almost identical atomoxetine studies that were designed to investigate the degree of ADHD-related difficulties, as perceived by patients, parents and physicians, in patients with ADHD treated with atomoxetine. These two instruments enabled us to assess ADHD-related difficulties a various times of the day, most importantly the evenings and the following mornings. This report is based on a secondary measure analysis of data from the two atomoxetine studies. The secondary measure analysis builds on two analyses for the primary endpoint (GIPD) based on two studies that were conducted separately for children and adolescents.

The two primary analyses have been published elsewhere [26,40]. The studies aimed to address the need for further research on response to psychopharmacological treatment in children and adolescents with ADHD [41]. Such research seems especially important because expectations in terms of response to treatment with stimulants versus non-stimulants and short-acting medications versus long-acting medications may differ between clinicians. Furthermore, there is a need for data in order to understand treatment variables such as dose frequency and symptom response over the day.

## Methods

### Study design and procedures

Patients were recruited from child and adolescent psychiatric and pediatric practices and outpatient clinics throughout Germany. Patients aged 6–17 years with ADHD as defined by the Diagnostic and Statistical Manual of Mental Disorders, Fourth Edition, Text Revision (DSM-IV-TR) [1] were eligible for one of the two studies. The diagnosis was confirmed using the "Diagnose-Checkliste Hyperkinetische Störungen" (DCL-HKS) (Diagnostic Checklist for Hyperkinetic Disorders) assessment tool, a structured instrument which is routinely used for diagnostic assessment of ADHD in Germany [42]. The items of this instrument correspond to those of the ADHD-RS [30,31]. Patients had to have an IQ of ≥ 70 based on the clinical judgment of the investigator. The exclusion criteria included clinically significant abnormal laboratory findings, acute or unstable medical conditions, cardiovascular disorder, history of seizures, pervasive developmental disorder, psychosis, bipolar disorder, suicidal ideation, any medical condition that might increase sympathetic nervous system activity, or the need for psychotropic medication other than study drug. Patients already being treated with atomoxetine were also excluded. The protocol was approved by an ethics committee and the study was conducted in accordance with the principles of the Declaration of Helsinki and international standards of Good Clinical Practice (GCP).

Following a wash-out period, baseline assessments were carried out with all instruments used. During the first week of treatment, patients received atomoxetine at a dose of approximately 0.5 mg/kg body weight (BW) per day. During the following 7 weeks, the recommended target dose was 1.2 mg/kg BW per day. This dose could be adjusted within a range of 0.5–1.4 mg/kg BW per day, depending on effectiveness and tolerability. Medication was given once a day in the morning. Assessments were carried out weekly during the first two weeks of treatment and every two weeks thereafter. After the 8-week treatment period, the physicians decided together with the patients and their parents whether the patient was to continue treatment for additional 16 weeks. Those who participated in this extension period continued on the same atomoxetine dose. Again, this dose could be adjusted within a range of 0.5–1.4 mg/kg BW per day as considered appropriate by the physician. During the extension period, three assessments were carried out: at 12, 16, and 24 weeks after baseline.

The following instruments were used: Weekly Rating of Evening and Morning Behavior – Revised (WREMB-R), Global Impression of Perceived Difficulties (GIPD), Attention-Deficit/Hyperactivity Disorder Rating Scale (ADHD-RS), and the Clinical Global Impression-Severity (CGI-S) scale. The data from both studies were combined and analyzed together.

The WREMB-R-Inv scale is based on the Daily Rating of Evening and Morning Behavior – Revised (DPREMB-R) scale [17]. The DPREMB-R has been validated for the assessment of ADHD-related behaviors [37] and has been used in several studies to assess behavior in children and adolescents with ADHD [17,43]. The original DPREMB-R has been modified to allow a weekly assessment of behavioral symptoms. This modified version of the scale has been used in a previous atomoxetine study [38]. In our studies, the investigator-rated version of the WREMB-R was used. The investigator rating was based on information provided by the parent. The WREMB-R measures 11 specific morning or evening behaviors (e. g. getting up and out of bed, doing or completing homework, sitting through dinner). The evening and morning subscores comprise 8 and 3 items, respectively. The 11 items of the Weekly Rating of Evening and Morning Behavior – Revised (WREMB-R) scale are shown in Table 1. The possible score for each item ranges from 0 (no difficulty) to 3 (a lot of difficulty).

**Table 1:** The 11 items of the Weekly Rating of Evening and Morning Behavior – Revised (WREMB-R) scale [17]

**Late afternoon/evening items (evening subscore)**
Problems with homework/tasks

Difficulty sitting through dinner

Difficulty playing quietly in the afternoon/evening

Inattentive and distractable in the afternoon/evening

Difficulty transitioning

Arguing or struggling in the afternoon/evening

Difficulty settling at bedtime

Difficulty falling asleep

**Early morning items (morning subscore)**

Difficulty getting out of bed

Difficulty getting ready

Arguing or struggling in the morning

The Global Impression of Perceived Difficulties (GIPD) instrument is a five-item rating of ADHD-related difficulties that assesses difficulties in the morning, during school, during homework, in the evening, and overall difficulties over the entire day and the night [26,40]. Each item is rated on a seven point scale (1 = not at all difficult, 7 = extremely difficult) and reflects the situation during the past week. This instrument was devised and validated to capture the patient's ADHD-related difficulties from a patient, parent (or primary caregiver), and physician perspective [39]. For the patient rating, an independent person (e. g. a study nurse) was allowed to provide assistance if the child was unable to fill in the scale on his/her own. The GIPD was designed to reflect any kind of difficulty perceived as such by the rater (patient, parent, physician). Thus, the scale captures ADHD-related difficulties in a very general and inclusive way, potentially including symptom-related difficulties, dysfunction, impairment, and subjective dissatisfaction.

The GIPD total score was calculated for each rater as the mean of the item scores ranging from 1 to 7. If one item was missing, the total score was also considered to be missing. The Attention-Deficit/Hyperactivity Disorder Rating Scale-IV-Parent Version: Investigator-Administered and Scored (ADHD-RS) is an 18-item scale, with one item for each of the 18 ADHD symptoms listed in DSM-IV-TR [30,31]. There are two subscales: the "hyperactivity/impulsivity" subscale is the sum of the even items, and the "inattention" subscale is the sum of the odd items. This scale is scored by an investigator while interviewing the parent (or primary caregiver). Reliability and validity of this scale has been demonstrated in several European samples, including Germany [44].

The Clinical Global Impression-Severity-Attention-Deficit/Hyperactivity Disorder (CGI-S ADHD) scale is a seven point single-item rating scale of the clinician's assessment of the severity of ADHD symptoms [32,33].

### Sample size and statistical analysis

Details on the sample size calculation for the two studies first using the GIPD have been published elsewhere in detail [26,40]. In summary, the sample sizes were sufficient to estimate the intraclass kappa of the GIPD for the different perspectives in sufficient precision using a 95% confidence interval. The data of all patients were evaluated (Full Analysis Set, FAS) using SAS version 8.0. The dataset for all analyses of changes from baseline to endpoint comprised the data of all patients with a baseline measurement and at least one post-baseline measurement during the 8 week treatment phase.

Evaluation was largely descriptive. To present WREMB-R and GIPD scores over time, a last observation carried forward (LOCF) approach was used. Two-sided confidence intervals (CI) were computed using a 95% confidence level. All inferences regarding statistical significance were based on comparisons of the 95% CI. This is equivalent to significance tests with p-values and a two-sided α-level of 5%.

The WREMB-R morning and evening subscores were compared with the corresponding GIPD items for morning and evening behavior by calculating Pearson's correlation coefficients with 95% CIs for each perspective, at each time point, and for all time points pooled. To avoid correlations of imputed values, only observed cases (OC) were used for these correlation analyses. There was no imputation of missing values. 95% confidence intervals for the correlation coefficients were computed based on Fisher's z-transformation.

## Results

### Patient population and disposition

**Figure 1:**
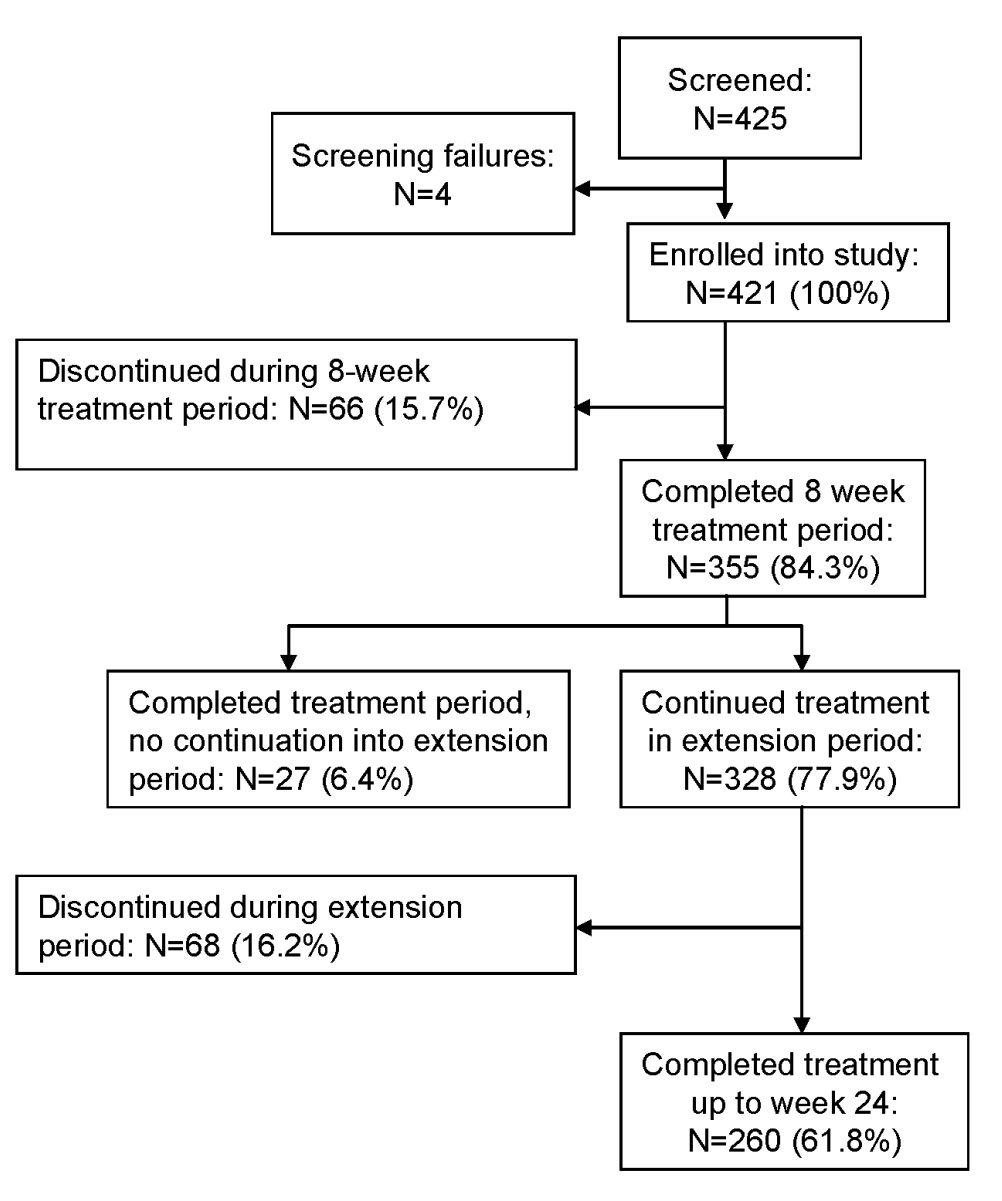
**Patient disposition**.

Overall, 421 patients (100%) diagnosed with ADHD according to DSM-IV-TR criteria were enrolled in the two studies and treated with atomoxetine [26,40]. Of these patients, 355 (84.3%) completed the initial 8-week treatment period, and 260 (61.8%) patients completed the extension period until week 24. Reasons for discontinuation were lack of efficacy (12.4%), parent decision (6.9%), adverse event (4.8%), protocol violation (3.6%), patient decision (2.4%), entry criteria exclusion (0.7%), physician decision (0.7%), and patient lost to follow-up (0.5%). The patient disposition is shown in detail in Figure 1.

**Figure 2:**
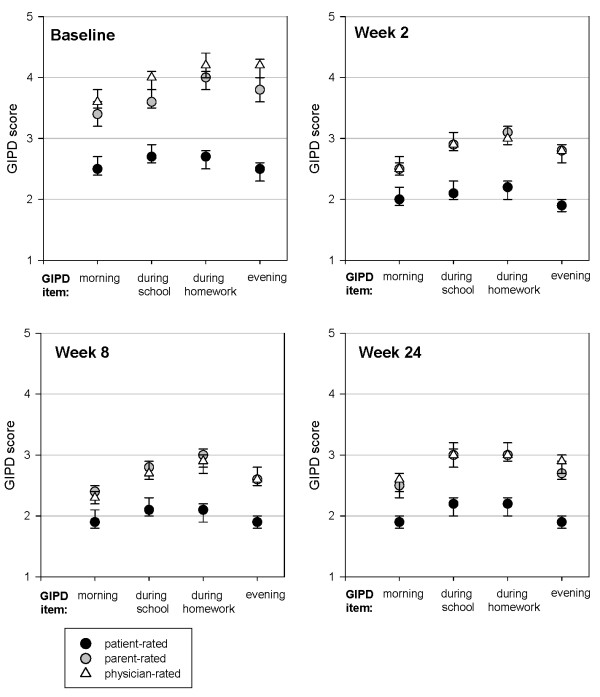
**GIPD scores for difficulties in the morning, during school, during homework and in the evening, as rated by patient, parent and physician at various time points (means and 95% CIs, LOCF data)**.

The baseline characteristics of the patients are shown in Table 2. At baseline, 78% of patients were rated as being at least "markedly ill" on the CGI-S scale for ADHD. Their mean ADHD-RS total score was 32.6 (± 10.9). On the GIPD-scale, patients perceived ADHD-related difficulties as being significantly less severe than parents and physicians did (see Table 2), as shown by the non-overlapping 95% confidence intervals (as shown in Figure 2).

**Table 2:** Patient characteristics (all patients, N = 421)

**Demographics**	
Boys, N (%)	338 (80.3)
Girls, N (%)	83 (19.7)
Age [years], mean (± SD)	11.1 (2.7)
**ADHD subtype, N (%)**	
Combined	278 (66.0)
predominantly inattentive	124 (29.5)
predominantly hyperactive-impulsive	6 (1.4)
ADHD, not otherwise specified	13 (3.1)
**Most frequent psychiatric comorbidities, N (%)**	
Conduct disorder	83 (19.7)
Oppositional defiant disorder	74 (17.6)
Enuresis	18 (4.3)
Emotional disorder of childhood	11 (2.6)
Tic disorder	10 (2.4)
Depression	6 (1.4)
Depressed mood	5 (1.2)
**Baseline disease characteristics, mean (± SD)**	
ADHD-RS, total score	32.6 (10.9)
CGI-Severity (of ADHD)	5.0 (0.8)
WREMB-R, total score	18.0 (7.0)
GIPD, total score patient-rated	13.1 (5.8)
GIPD, total score parent-rated	18.6 (6.1)
GIPD, total score physician-rated	20.3 (5.9)

Abbreviations: ADHD = Attention deficit/hyperactivity disorder, ADHD-RS = ADHD Rating Scale (parent-rated, investigator-administered and scored), CGI-Severity = Clinical Global Impression, Severity, GIPD = Global Impression of perceived difficulties, SD = standard deviation, WREMB-R = Weekly Ratings of Evening and Morning Behavior-Revised

In boys, a combined subtype of ADHD according to DSM-IV criteria was diagnosed most frequently (N = 239, 70.7%) followed by the predominantly inattentive subtype (N = 86, 25.4%). The combined subtype of ADHD according to DSM-IV criteria was diagnosed in 39 girls (47.0%) and the predominantly inattentive subtype in 38 girls (45.8%). The subgroups of patients with "predominantly hyperactive-impulsive subtype" and "ADHD, not otherwise specified" were small (6 and 13 individuals, respectively).

The most frequent pre-existing comorbid conditions in the two study populations were psychiatric comorbidities (Table 2). 349 (82.9%) of patients had previously received a medication for ADHD. Medications most frequently used prior to entering the study were short-acting methylphenidate (N = 290, 68.9%), long-acting methylphenidate (N = 196, 46.6%), amphetamines (N = 56, 13.3%), antipsychotic drugs (N = 12, 2.9%) and herbal/complementary therapies (N = 10, 2.4%). The most frequent reason for discontinuation of previous treatment prior to entering the study was inadequate response (N = 216, 51.3%). N = 68 patients (16.2%) discontinued the previous treatment because of adverse events.

The mean atomoxetine dose given during the first week of treatment was 0.5 mg/kg body weight (BW) per day (SD 0.07, range 0.4 – 0.8 mg/kg BW per day). Thereafter, the mean dose for the respective visit intervals ranged between 1.17 and 1.18 mg/kg BW per day (range 0.4 – 1.5 mg/kg BW per day).

Concomitant medication was taken by 272 (64.6%) of the patients. Cough and cold remedies, analgesics, antibiotics and herbal/complementary medicines were given most frequently. Concomitant behavioral therapy was given to 27 (6.4%) patients, and 20 (4.8%) patients received additional occupational therapy.

### WREMB-R evening and morning subscores

**Figure 3:**
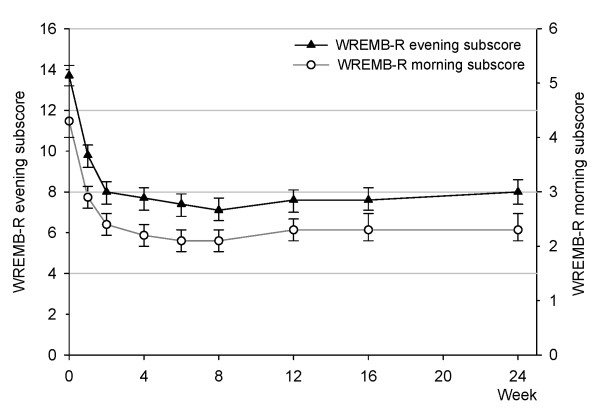
**WREMB-R evening and morning subscores over time (means and 95% CIs, LOCF data)**. The two scalings reflect the different number of items of the evening (8 items) and morning (3 items) subscores.

Evening and morning behavioral symptoms, as reflected by the WREMB-R evening and morning subscores, both decreased significantly over time, as shown by non-overlapping confidence intervals (see Table 3 and Figure 3). The greatest change occurred within the first two weeks, after which the scores remained relatively stable until the end of study (24 weeks). The mean evening subscore (95% CI) decreased significantly from 13.7 (13.2 to 14.2) at baseline down to 8.0 (7.4 to 8.5) at week 2 and remained at 8.0 (7.4 to 8.6) until week 24. Mean morning subscores decreased significantly from 4.3 (4.0 to 4.5) down to 2.4 (2.2 to 2.6) at week 2 and remained at 2.3 (2.1 to 2.6) until week 24. Figure 3 shows the two WREMB-R subscores over time, scaling was adjusted to reflect the different number of items, 8 items for the evening subscore and 3 items for the morning subscore. Nevertheless, the WREMB-R evening subscores were higher than the respective morning subscores at each point in time from baseline until Week 24 (Figure 3).

**Table 3:** WREMB-R evening and morning subscores and GIPD single item scores (mean ± SD, LOCF).

	**Baseline (BL)**		**Week 2**			**Week 8**			**Week 24**		
	
	**N**	**Score**	**N**	**Score**	**Change**	**N**	**Score**	**Change**	**N**	**Score**	**Change**
**WREMB-R**											
Evening subscore (8 items)	418	13.7 ± 5.24	419	8.0 ± 5.57	-5.7 ± 5.43	419	7.1 ± 5.78	-6.6 ± 6.14	419	8.0 ± 6.16	-5.7 ± 6.30
Morning subscore (3 items)	419	4.3 ± 2.49	418	2.4 ± 2.23	-1.8 ± 2.53	419	2.1 ± 2.22	-2.2 ± 2.84	419	2.3 ± 2.37	-1.9 ± 2.83
**GIPD**											
											
Difficulties in the morning											
patient-rated	418	2.5 ± 1.60	418	2.0 ± 1.40	-0.5 ± 1.74	419	1.9 ± 1.35	-0.6 ± 1.80	419	1.9 ± 1.40	-0.6 ± 1.68
parent-rated^a^	418	3.4 ± 1.69	393	2.5 ± 1.44	-0.9 ± 1.77	404	2.4 ± 1.55	-1.0 ± 1.97	409	2.5 ± 1.62	-0.9 ± 1.97
physician-rated	419	3.6 ± 1.58	419	2.5 ± 1.33	-1.1 ± 1.71	419	2.3 ± 1.45	-1.3 ± 1.84	419	2.6 ± 1.57	-1.1 ± 1.82
Difficulties during school											
patient-rated	417	2.7 ± 1.65	417	2.1 ± 1.34	-0.6 ± 1.66	418	2.1 ± 1.52	-0.6 ± 1.93	418	2.2 ± 1.60	-0.5 ± 2.04
parent-rated^a^	415	3.6 ± 1.58	389	2.9 ± 1.48	-0.8 ± 1.75	403	2.8 ± 1.61	-0.9 ± 1.96	408	3.0 ± 1.73	-0.7 ± 2.11
physician-rated	416	4.0 ± 1.51	419	2.9 ± 1.40	-1.1 ± 1.77	419	2.7 ± 1.60	-1.3 ± 2.00	419	3.0 ± 1.75	-1.0 ± 2.12
Difficulties during homework											
patient-rated	416	2.7 ± 1.67	417	2.2 ± 1.51	-0.5 ± 1.81	418	2.1 ± 1.50	-0.6 ± 1.91	418	2.2 ± 1.59	-0.5 ± 1.93
parent-rated^a^	417	4.0 ± 1.65	390	3.1 ± 1.51	-1.0 ± 1.67	403	3.0 ± 1.63	-1.0 ± 1.97	408	3.0 ± 1.69	-1.0 ± 2.03
physician-rated	415	4.2 ± 1.56	418	3.0 ± 1.46	-1.2 ± 1.72	419	2.9 ± 1.62	-1.4 ± 1.91	419	3.0 ± 1.68	-1.2 ± 1.99
Difficulties in the evening											
patient-rated	418	2.5 ± 1.72	419	1.9 ± 1.26	-0.6 ± 1.67	419	1.9 ± 1.32	-0.6 ± 1.86	419	1.9 ± 1.34	-0.6 ± 1.84
parent-rated^a^	419	3.8 ± 1.60	394	2.8 ± 1.50	-1.0 ± 1.66	404	2.6 ± 1.52	-1.2 ± 1.79	409	2.7 ± 1.60	-1.1 ± 1.87
physician-rated	419	4.2 ± 1.47	419	2.8 ± 1.43	-1.4 ± 1.56	419	2.6 ± 1.54	-1.5 ± 1.82	419	2.9 ± 1.72	-1.3 ± 1.85

Change is shown relative to baseline.

BL = baseline, GIPD = Global Impression of Perceived Difficulties, LOCF = last patient carried forward analysis, N = Number of patients, SD = standard deviation, WREMB-R = Weekly Ratings of Evening and Morning Behavior-Revised.

^a^ Post-baseline parent-rated GIPD scores were considered only if rated by the baseline rater.

### GIPD scores at different times of the day, as perceived by patients, parents and physicians

All four GIPD items reflecting ADHD-related difficulties in the morning, during school, during homework and in the evening improved over time (Table 3). For each item, the majority of change occurred within the first 2 weeks. Scores then remained stable until week 24. Figure 2 shows the ADHD-related difficulties at various times of the day as perceived from a patient, parent and physician perspective. At each time of the day and at all time points up to 24 weeks, patients rated the ADHD-related difficulties as being less severe than parents and physicians did. Parents and physicians tended to perceive the ADHD-related difficulties as being similar in the morning and the evening. Until week 8, parents and physicians rated the perceived difficulties as being most pronounced during homework. For example, mean parent ratings at baseline (95% CI) were 3.4 (3.2 to 3.6) for difficulties in the morning, 3.6 (3.5 to 3.8) for difficulties during school, 4.0 (3.8 to 4.1) for difficulties during homework, and 3.8 (3.6 to 4.0) in the evening. At week 2, all parent ratings had decreased significantly to 2.5 (2.4 to 2.7) for difficulties in the morning, 2.9 (2.8 to 3.1) for difficulties during school, 3.1 (2.9 to 3.2) for difficulties during homework, and 2.8 (2.6 to 2.9) for difficulties in the evening. Ratings then generally remained stable until week 24.

Patients did not only perceive their ADHD-related difficulties as being less severe than parents and physician, but also differed slightly in their perception of difficulties at various times of the day. As compared to parents and physicians, patients perceived their morning and evening difficulties as being similarly severe. At baseline, mean patient ratings (95% CI) were 2.5 (2.4 to 2.7) for difficulties in the morning and 2.5 (2.3 to 2.6) for difficulties in the evening. At week 2, both ratings had decreased significantly to 2.0 (1.9 to 2.2) for difficulties in the morning and 1.9 (1.8 to 2.0) for difficulties in the evening. Again, ratings then remained stable until week 24.

### Correlation of WREMB-R evening and morning subscores and the respective GIPD scores for the evening and morning items

As shown in Table 4, correlation between the WREMB-R evening and morning subscores and the investigator-rated GIPD scores for the evening and morning items was high for the GIPD parent ratings (e.g. for evening score, all visits pooled: 0.708, 95% CI: 0.690 to 0.725) and physician ratings (0.790, 95% CI: 0.776 to 0.803). At all time points as well as for all visits pooled, the correlation was significantly lower for ratings from the patient perspective vs. parent and physician perspective, as indicated by the non-overlapping 95% confidence intervals. For example, the correlation between the WREMB-R evening subscore and the GIPD score for the evening item (patient-rated, all visits pooled) was 0.351 (95% CI 0.320 to 0.382). This pattern was similar for all time points from baseline until week 24, with correlations slightly increasing over time (Table 4).

**Table 4:** Correlation of WREMB-R evening and morning subscores with GIPD scores for evening and morning items (Pearson's Correlation Coefficients and 95% CI; OC)

	**Correlation with WREMB-R evening subscore**					
	**GIPD evening (patient-rated)**		**GIPD evening (parent-rated)**		**GIPD evening (physician-rated)**	

**Week**	**N**	**Correlation**	**N**	**Correlation**	**N**	**Correlation**

0	416	0.321 [0.231–0.404]	413	0.537 [0.464–0.602]	414	0.695 [0.641–0.741]
1	412	0.276 [0.184–0.363]	401	0.625 [0.561–0.681]	410	0.717 [0.665–0.760]
2	397	0.337 [0.247–0.421]	392	0.686 [0.630–0.735]	399	0.790 [0.750–0.824]
4	383	0.348 [0.257–0.433]	382	0.672 [0.612–0.723]	387	0.767 [0.722–0.805]
6	365	0.398 [0.307–0.480]	359	0.774 [0.728–0.812]	368	0.790 [0.748–0.826]
8	326	0.270 [0.166–0.368]	324	0.731 [0.675–0.778]	326	0.772 [0.723–0.812]
12	314	0.177 [0.067–0.282]	312	0.684 [0.619–0.738]	317	0.707 [0.647–0.758]
16	271	0.347 [0.237–0.447]	268	0.683 [0.612–0.741]	274	0.764 [0.709–0.809]
24	259	0.336 [0.223–0.440]	257	0.733 [0.670–0.784]	261	0.767 [0.711–0.812]
all visits pooled	3143	0.351 [0.320–0.382]	3108	0.708 [0.690–0.725]	3156	0.790 [0.776–0.803]

	**Correlation with WREMB-R morning subscore**					
	
	**GIPD morning (patient-rated)**		**GIPD morning (parent-rated)**		**GIPD morning (physician-rated)**	
	
	**N**	**Correlation**	**N**	**Correlation**	**N**	**Correlation**

0	416	0.258 [0.166–0.346]	413	0.612 [0.547–0.668]	414	0.714 [0.663–0.758]
1	411	0.442 [0.361–0.517]	400	0.644 [0.582–0.698]	409	0.727 [0.677–0.769]
2	397	0.318 [0.226–0.403]	392	0.699 [0.644–0.746]	399	0.760 [0.714–0.798]
4	383	0.311 [0.217–0.398]	382	0.754 [0.706–0.794]	387	0.742 [0.693–0.783]
6	365	0.297 [0.200–0.388]	359	0.738 [0.686–0.781]	368	0.778 [0.734–0.815]
8	326	0.331 [0.231–0.424]	324	0.677 [0.612–0.731]	326	0.776 [0.729–0.816]
12	314	0.332 [0.229–0.426]	312	0.734 [0.678–0.781]	317	0.791 [0.746–0.829]
16	271	0.361 [0.252–0.460]	268	0.710 [0.645–0.764]	274	0.779 [0.727–0.821]
24	259	0.313 [0.198–0.418]	257	0.734 [0.671–0.785]	261	0.778 [0.725–0.822]
all visits pooled	3142	0.371 [0.341–0.401]	3107	0.719 [0.701–0.735]	3155	0.786 [0.772–0.799]

CI = confidence interval, GIPD = Global Impression of Perceived Difficulties, OC = observed cases analysis, WREMB-R = Weekly Ratings of Evening and Morning Behavior-Revised.

### Tolerability

Treatment emergent adverse events were reported in 331 (78.6%) patients over the entire study period. Adverse events reported in more than 5% of all patients (N = 421, 100%) were: fatigue 106 (25.2%), headache 86 (20.4%), nausea 77 (18.3%), vomiting 52 (12.4%), upper abdominal pain 42 (10.0%), nasopharyngitis 41 (9.7%), decreased appetite 30 (7.1%), diarrhea 27 (6.4%), abdominal pain 24 (5.7%), upper respiratory tract infection 23 (5.5%), cough 21 (5.0%), dizziness 21 (5.0%). In 207 (49.2%) patients the investigator considered the adverse event possibly related to atomoxetine. Adverse events reported in more than 5% of the patients and rated as possibly related to atomoxetine were: fatigue (N = 94, 22.3%), nausea (N = 57, 13.5%), headache (N = 36, 8.6%), upper abdominal pain (N = 30, 7.1%), reduced appetite (N = 26, 6.2%), and vomiting (N = 21, 5.0%). There were 11 (2.6%) patients with serious adverse events: dissociation, fall, fatigue and forearm fracture were reported twice, abdominal injury, abdominal pain, alcohol poisoning, appendicitis, circulatory collapse, depression, disturbance in attention, dizziness, drug abuse, head injury, hypothermia, injury, somnolence, tendon rupture, vasoconstriction, and vomiting were reported once (several patients experienced more than one serious adverse event). In two of these 11 patients, the adverse events were considered related to atomoxetine by the physician (one patient with vomiting and one patient with peripheral vasoconstriction, attention disturbance, fatigue, dizziness, abdominal pain and feeling of absence). Overall, no clinically relevant changes in vital signs were observed for the entire sample.

## Discussion

The aim of this secondary measure analysis was to evaluate whether behavioral symptoms and ADHD-related difficulties during treatment with atomoxetine as perceived by patients, parents and physicians, differ at various times of the day. A total of 421 children and adolescents diagnosed with ADHD according to DSM-IV criteria were included in this analysis of data from two open-label studies [26,40]. The patient characteristics of this sample (Table 2) closely resemble those of previous atomoxetine studies in children and adolescents with ADHD, which facilitates comparison between studies.

The WREMB-R evening and morning scores decreased over the 24-week observation period (Table 3). This finding which is based on two open label studies is consistent with the results of two previous randomized double blind studies that assessed evening and morning behavior in children treated with atomoxetine [17,23] and showed that the greatest change seems to occur during the first few weeks of treatment. As in these two previous studies, the improvement in terms of morning and evening behavior in this study persisted until the end of the 24-week observation period. These findings are in line with findings from a double-blind, placebo-controlled atomoxetine study using the ADHD Rating Scale (ADHD-RS) to measure core symptom efficacy and investigate the onset of action [43].

At baseline, the parent- and physician rated GIPD scores over the day (morning, during school, during homework, evening) ranged between 3.4 and 4.2 (3 = a little difficult, 4 = moderately difficult). Parent and physician rated scores were highest during homework, lower during school and in the evening, and lowest in the mornings (Figure 2). Patients rated their difficulties as significantly less severe, and the pattern of symptoms over the day was less pronounced. Significant decreases changes over time were shown for all four items of the GIPD (Table 3, Figure 2), and for all three rater perspectives. These findings suggest that ADHD-related difficulties are present at various times of the day and that these difficulties respond to treatment.

Interestingly, the two GIPD items that reflect ADHD-related difficulties in the evening and in the morning improved in a pattern similar to the WREMB-R evening and morning subscores (Table 3). This held true from all three perspectives (patient, parent, physician). For both the evening and morning items, the majority of change occurred within the first 2 weeks, with scores remaining stable until week 24 (Table 3). Thus, the results obtained using the morning and the evening items of the GIPD are consistent with those obtained using the WREMB-R.

Correlation between the investigator-rated WREMB-R evening and morning subscores and the corresponding GIPD scores for the evening and morning items as rated by parents and physicians was high (Table 4). The correlations were significantly lower using GIPD ratings from the patient perspective. This pattern was found for all time points from baseline until week 24, with correlations increasing slightly over time (Table 4). Obviously, children and adolescents perceived their difficulties as being significantly less severe at all times of the day throughout the study. This suggests that children and adolescents may recognize or report their ADHD-related difficulties to a smaller degree than the adult raters (parents, physicians). Other studies on correlations between ratings by parents and children (or adolescents) show little agreement [45]. Thus, the similar discrepancy in the two studies reported here is not surprising. However, the higher correlation of the parent and physician perspective may also be due to the physicians basing their ratings primarily on the information provided by the parents rather than the information provided by the patients. In summary, our findings suggest that although there are differences in the degree of difficulties perceived from the three perspectives (patients, parents, physicians), all see an improvement of perceived ADHD-related difficulties over time.

In the two studies reported here, discontinuation rates were low [26,40]. The three most common reasons for discontinuation were lack of efficacy (12.4%), parent decision (6.9%), and adverse events (4.8%). The adverse event profile found in the two studies was very similar to those reported in randomized, placebo-controlled trials [17,46,47]. The two most common AEs possibly related to atomoxetine were fatigue (22.3%) and nausea (13.5%). Both are well-known potential adverse reactions to atomoxetine.

These studies and analyses have several limitations. Most importantly, they did not include a placebo control, so that the degree to which the results reflect drug-specific effects cannot be determined definitively. Due to the open-label design, unspecific factors such as rater bias, expectation effects, and time effects cannot be ruled out. However, this does not automatically compromise the validity of the results [48]. Also, sensitivity regarding differences between placebo and active comparator cannot be determined. A further limitation of this study is the age-distribution of the sample that does not reflect the age-distribution of individuals with ADHD in the general population. This is due to the fact that this analysis is based on two identical studies, one in children and one in adolescents.

## Conclusion

Overall, findings from this secondary measure analysis of two open-label studies suggest that morning and evening behavior, as reflected by the WREMB-R, and ADHD-related difficulties in the mornings and evenings, as reflected by the GIPD, improve over time with once daily atomoxetine treatment. This effect persisted over a period of up to 24 weeks. This finding applied to all three rater perspectives: patient, parent and physician. The value of using instruments such as the WREMB-R and the GIPD in a clinical context is that they can guide the physician in working towards remission, not only in terms of core symptom response, but also in terms of subjective patient outcome.

## Competing interests

PMW, AS, and KHE are full-time employees of Lilly Deutschland, RWD is a former employee of Lilly Deutschland and now holds a Eli Lilly Endowed Chair of Pediatric Psychopharmacology. PMW and RWD own Eli Lilly & Co. stock. GL has received research grants and speaker honoraria from Eli Lilly & Co. and is member of a Lilly Advisory Board.

## Authors' contributions

PMW, RWD and AS developed the two clinical trials. AS developed the analyses reported in this manuscript. All authors participated in development of the GIPD scale and the interpretation of data. PMW, AS and KHE drafted the manuscript, RWD and GL revised it critically for important intellectual content. All authors read and approved the final manuscript.
